# A New Remote Sensing-Based System for the Monitoring and Analysis of Growth and Gas Exchange Rates of Photosynthetic Microorganisms Under Simulated Non-Terrestrial Conditions

**DOI:** 10.3389/fpls.2020.00182

**Published:** 2020-03-04

**Authors:** Mariano Battistuzzi, Lorenzo Cocola, Bernardo Salasnich, M. Sergio Erculiani, Eleonora Alei, Tomas Morosinotto, Riccardo Claudi, Luca Poletto, Nicoletta La Rocca

**Affiliations:** ^1^ Centro di Ateneo di Studi e Attività Spaziali (CISAS) “Giuseppe Colombo”, Padova, Italy; ^2^ Department of Biology, University of Padova, Padova, Italy; ^3^ CNR, Institute for Photonics and Nanotechnologies, Padova, Italy; ^4^ INAF, Astronomical Observatory of Padova, Padova, Italy

**Keywords:** photosynthesis, cyanobacteria, simulation chamber, remote sensing, reflectance spectra, Normalized Difference Vegetation Indexes

## Abstract

Oxygenic photosynthetic microorganisms are a focal point of research in the context of human space exploration. As part of the bioregenerative life-support systems, they could have a key role in the production of breathable O_2_, edible biomasses and in the regeneration of CO_2_ rich-atmospheres and wastewaters produced by astronauts. The test of the organism’s response to simulated physico-chemical parameters of planetary bodies could also provide important information about their habitability potential. It is believed that the success of future planetary and space missions will require innovative technologies, developed on the base of preliminary experiments in custom-made laboratory facilities. In this context, simulation chambers will play a pivotal role by allowing the growth of the microorganisms under controlled conditions and the evaluation in real-time of their biomass productivity and impact on atmosphere composition. We here present a system capable of addressing these requirements with high replicability and low costs. The setup is composed by three main parts: 1) a Star Light Simulator, able to generate different light intensities and spectra, including those of non-solar stars; 2) an Atmosphere Simulator Chamber where cultures of photosynthetic microorganisms can be exposed to different gas compositions; 3) a reflectivity detection system to measure from remote the Normalized Difference Vegetation Indexes (NDVI). Such a setup allows us to monitor photosynthetic microorganism’s growth and gas exchange performances under selected conditions of light quality and intensity, temperature, pressure, and atmospheres simulating non-terrestrial environments. All parameters are detected by remote sensing techniques, thus without interfering with the experiments and altering the environmental conditions set. We validated the setup by growing cyanobacteria liquid cultures under different light intensities of solar illumination, collecting data on their growth rate, photosynthetic activity, and gas exchange capacity. We utilized the reflectivity detection system to measure the reflection spectra of the growing cultures, obtaining their relative NDVI that was shown to correlate with optical density, chlorophyll content, and dry weight, demonstrating the potential application of this index as a proxy of growth.

## Introduction

Oxygenic photosynthetic microorganisms such as cyanobacteria and eukaryotic algae have been fundamental for life evolution on our planet by creating an oxygen-rich atmosphere. They still play a fundamental role in supporting lifeforms on Earth, being responsible for approximately 50% of global oxygen production (Geider et al., 2001) and being at the base of the food chain as primary producers (Fischer et al., 2016; Dick et al., 2018). Not surprisingly, considering such a role, cyanobacteria, and microalgae are also considered key players to achieve sustainable bioregenerative life-support for human exploration of the space (Kitaya et al., 2005; Hu et al., 2012; Li et al., 2013). Bioregenerative life support systems (BLSSs) are in fact developed with the aim of continuously recycling resources *via* metabolic/biological processes, in order to minimize logistics and re-supply from the Earth. So far, only seven facilities have been realized worldwide, capable of integrating full-scale life support system tests [reviewed by (Escobar and Nabity, 2017)]. They generally consist of human/microbial/plant associations where microorganism strains are specifically selected from wild type or engineered species to obtain reliable culturing and high rate processes. These facilities, as in the case of the MELISSA (Micro-Ecological Life Support System Alternative) program, could include specific modules for the cultivation of selected cyanobacteria or microalgae (Poughon et al., 2009). Confined environments like the International Space Station (ISS) or future terraforming structures on planetary bodies, e.g., the Moon or Mars, could benefit from oxygenic photosynthetic organisms due to their ability to produce biomass autotrophically, to scrub CO_2_ from the crew cabin thereby maintaining a habitable atmospheric composition and to recycle wastewaters and human products (Tikhomirov et al., 2007; Verseux et al., 2016). Photosynthetic microorganisms such as plants are oxygen producers and their biomass is a valuable feedstock for the sustainable production of several bio-commodities. However, with respect to plants, they have simpler requirements for growth, and they are more suitable for cultivation in confined spaces. Furthermore, cyanobacteria and microalgae are also able to grow on wastewater and at higher CO_2_ concentrations (Bugbee et al., 1994; Wu et al., 2008; Molazadeh et al., 2019). A few genera that have been assessed for these applications are *Arthrospira* spp. between cyanobacteria (Travieso et al., 2001; Gòdia et al., 2002; Hu et al., 2012) and *Chlorella* spp. among microalgae (Escobar and Nabity, 2017). Nevertheless, many other cyanobacteria and microalgae species are highly efficient in converting solar energy, CO_2_, H_2_O, and mineral nutrients in O_2_ and biomasses, also providing a source of high-value compounds, such as antioxidants pigments, essential fatty acids, and amino acids (Wijffels et al., 2013; Leu and Boussiba, 2014). The identification of the most suitable strains of photoautotrophic oxygenic microorganisms to be used as players within life support systems requires a better understanding of their physiological responses under non-terrestrial simulated conditions, characterized by physico-chemical parameters different from terrestrial ones, such as ionizing radiations and peculiar light intensities and spectra, gas compositions, and temperatures. However, the above mentioned full-scale integrated facilities are too large, expensive, time, and labor-consuming to obtain experimental replicates, needed to predict their performances by dedicated mathematical models (Escobar and Nabity, 2017). Thus, the possibility to analyze, with high controllability, replicability, and low costs, the metabolic processes of cyanobacteria or microalgae in closed systems simulating space or extra-terrestrial environmental parameters, represents an important step to develop the technologies to face the challenge of human exploration beyond the Earth’s boundaries. The study of oxygenic photosynthetic microorganisms’ responses to simulated exoplanets’ physico-chemical parameters (at least the already known or modelized) could also provide important information about the habitability potential of extraterrestrial planets. For example, the impact of their photosynthetic gas exchange on simulated atmospheres could provide a database of atmospheric biosignatures to be compared with future astronomical observations, such as those based on spectrometers mounted on large ground telescopes, like HIRES at the European extremely large telescope (E-ELT) (Marconi et al., 2016), or on the next generation satellites [JWST, ARIEL, (Stevenson et al., 2016; Pascale et al., 2018)], designed to characterize exoplanet’s atmospheres. In this context, cyanobacteria are considered the most promising organisms to be tested due to the capability of several species to thrive even in extreme environments of the Earth, such as hot and cold deserts and polar ices, often considered as analogs for extraterrestrial planets. Many strains are also able to perform N_2_ fixation and others have been shown durable in atmospheres with high CO_2_ concentrations, even up to 100% after gradual adaptation (Allakhverdiev et al., 2009; Masukawa et al., 2012; Murukesan et al., 2016). Some cyanobacteria have been furthermore demonstrated to be able to withstand space conditions when protected from UV-radiation (Billi et al., 2013). Several rocky Earth-like planets have been recently found orbiting the habitable zone of M-dwarf stars, the lowest in mass and irradiance and the most common type of stars in our galaxy (about 76% of total stars). These stars are considered excellent candidates for terrestrial planet searches, as they can live long enough to allow for life evolution. However, M-dwarfs stars are characterized by a very low irradiance, particularly in the visible part of the spectrum, with a major component in the far red (FR) and infrared (IR) wavelengths, compared to the Sun. This irradiation with less energy is expected to be challenging for organisms performing oxygenic photosynthesis. However, among cyanobacteria biodiversity there are species able to extend the wavelengths of operational photosynthesis up to 750 nm thanks to the ability to accumulate peculiar pigments, chlorophylls *d* and *f* (Gan and Bryant, 2015). This opens new possibilities to assess whether oxygenic photosynthesis as we know it could be possible under M-dwarf starlight spectra. Such capacity was so far only hypothesized (Gale and Wandel, 2017; Takizawa et al., 2017; Ritchie et al., 2018; Wandel, 2018), but never tested by simulation experiments. Here we present an experimental setup allowing to grow photosynthetic microorganisms under different environmental conditions, including simulated non-terrestrial atmospheres and lights. The setup is also equipped to control and regulate temperature and pressure and to simultaneously detect through remote sensors (without disturbing the chamber environment) CO_2_ consumption and O_2_ evolution as well as growth of the photosynthetic microorganisms directly exposed to the selected growth conditions. The equipment is composed of three main instruments: 1) a Star Light Simulator (SLS) capable of generating different light intensities and spectra, including those of simulated warm to cold stars (F/G/K/M); 2) an atmosphere simulator chamber (ASC) where cultures of photosynthetic microorganisms can be grown under selected atmospheric gas composition and different light regimes; 3) a newly developed reflectivity detection system (RDS) to measure from remote the NDVI parameter as a proxy of the culture growth. We also present here the validation of the setup and its use for the cultivation of the Cyanobacterium *Synechocystis* sp. PCC6803, a model organism for photosynthesis and astrobiology researches (Murukesan et al., 2016).

## Materials and Methods

### Setup Description

#### Star Light Simulator

The Star Light Simulator (SLS) is an innovative device [described in (Erculiani et al., 2016; Trivellin et al., 2016; Salasnich et al., 2018)] capable of simulating the emission spectra of different kinds of warm to cold stars (F/G/K/M) as well as emitting single wavelength lights, with a great flexibility based on the scientific goals required. The main concept behind the device is that of modularity: it is composed of a copper ring on which different printed circuit boards are combined forming a mosaic of circuits. On them, 273 air-cooled diodes are soldered, divided into 25 current controlled channels. Direct emission channels are: 365 nm (LED Engin LZ1-00U600), 380 nm (Lumileds LHUV-0380-0200), 405 nm (Lumileds LHUV-0400-0500), 425 nm (Lumileds LHUV-0420-0650), 448 nm (Lumileds LXZ1-PR01), 470 nm (Lumileds LXZ1-PB01), 485 nm (Osram CRBP-HXJX-47-1), 505 nm (Lumileds LXZ1-PE01), 530 nm (Lumileds LXZ1-PM01), 568 nm (Lumileds LXZ1-PX01), 590 nm (Lumileds LXZ1-PL01), 627 nm (Lumileds LXZ1-PD01), 655 nm (Lumileds LXZ1-PA01), 680 nm (Roithner SMB1N-680), 700 nm (Roithner SMB1N-700), 720 nm (Roithner SMB1N-720D), 740 nm (LED Engin LZ1-00R300), 760 nm (Roithner SMB1N-760D), 780 nm (Roithner SMB1N-780D), 810 nm (Roithner SMB1N-810D), 830 nm (Roithner SMB1N-830N), 870 nm (Osram SFH 4715S), 880 nm (Roithner SMB1N-880), 940 nm (Osram SFH 4725S); one channel is driving white 2780K LEDs (Lumileds 997-LXZ1-2280-5-2200). The SLS is directly interfaced with a PC and dedicated software (Salasnich et al., 2018). Star spectra can be loaded from the internal database, but each LED channel can be turned on or off independently and its intensity can be trimmed according to necessity. The light emitted from the instrument is monitored and corrected thanks to a spectrometer (Flame, Ocean Optics), this enables accurate simulation of the desired spectra during laboratory experiments.

#### Atmosphere Simulator Chamber

**Figure 1:**
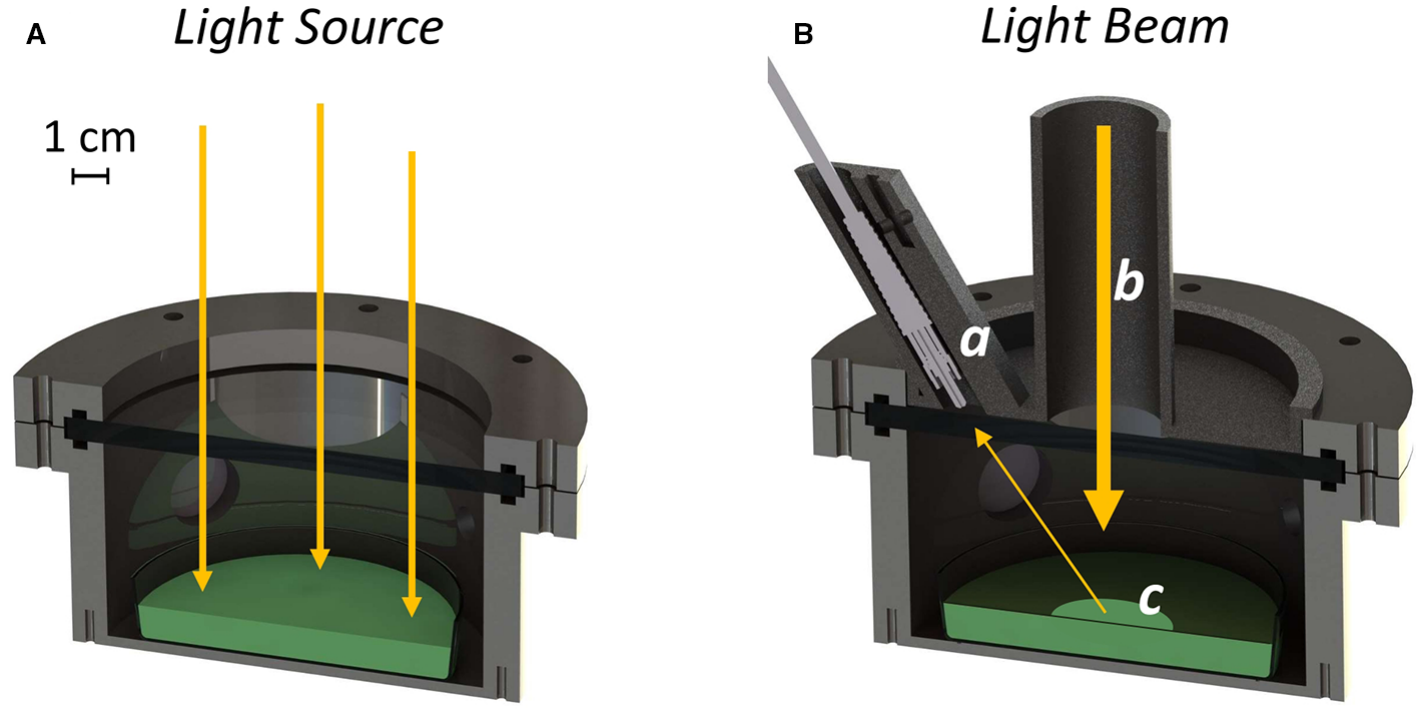
Rendering of the experimental setup in the “growth” configuration **(A)** and during the reflectance measurement **(B)**. The optical fiber is mounted inside the baffling system (a). The light coming from the Star Light Simulator is collimated through the baffling system (b), in a way that leaves only a little portion of the culture exposed to the light (c).

The atmosphere simulator chamber (ASC) is based on the experience of the miniLISA chamber (Claudi et al., 2015; Erculiani et al., 2016) with some modifications. The herein presented device is built around an environmental growth chamber with a volume of 0.5 L designed to grow photosynthetic microorganisms in open glass Petri dishes (**Figure 1A**). The experimental vessel is made of stainless steel with a top Borofloat window, that allows the illumination of the samples by the SLS or other light sources. The material is transparent to all the spectral ranges useful for the studies. Four 2.5 cm wedge windows are available on the sides for gas diagnostics. Two of the windows are used by the device for carbon dioxide sensing, which uses tunable diode laser absorption spectroscopy (TDLAS) in the 2-µm wavelength region (Danilović et al., 2016; Ghetti et al., 2018). Briefly, a diode laser is tuned around one of the absorption lines of carbon dioxide, at 2.04 µm. The laser beam enters the cell through an optical window and crosses the cell on a full diameter, which is the optical path where absorption is measured. A photo-detector, placed in close contact on the output window, measures the light absorption as a function of the scanned wavelengths, which is proportional to the carbon dioxide concentration. Oxygen concentration is sensed with a commercial oxygen analyzer (Nomasense O_2_ P300, Vinventions) based on fluorescence quenching. The instrument emits blue light through an optical fiber and into a sensor tablet attached to the inside of one of the ASC wedge windows. The sensor tablet absorbs blue light and shortly after emits red fluorescent light, that is sent back to the instrument. The readout device measures the amount of time that takes between the sending and the reception of the light, as it depends upon the concentration of oxygen surrounding the sensor. Data collected from both sensors were processed in MATLAB (MathWorks) to obtain the quantities of exchanged gas in micromoles by applying the ideal gas law. Inside the ASC different parameters like the temperature of the sample, the pressure, and the atmospheric composition can be tuned. The temperature of the sample is kept constant through a set of four Peltier cells (tec1-12706) placed on the bottom of the stainless vessel. An NTC thermistor is embedded in the cell bottom for the temperature control loop. The ASC is designed to work at temperatures ranging from 15 to 40°C. The vessel is provided with pipe fittings and connected to an array of flow meters and needle valves (each for a different input gas e.g., N_2_, O_2_, CO_2_) to inject atmospheres of arbitrary compositions. Pressure can be tuned from vacuum to 200 kPa and is monitored through a manometer (Druck dpi 104, General Electric). The SLS and the ASC are mounted in an outer temperature-controlled experimental box, which is required to keep a defined illumination geometry and to provide a constant temperature around the ASC. This resulted to be important not only for the accuracy of the ASC temperature control loop but also to avoid condensation on the optical windows of the chamber, that could influence the light transmission to the culture of photosynthetic microorganisms to be tested. This is achieved by keeping the outer temperature slightly higher (2–4°C) than the sample temperature.

#### Reflectivity Detection System

The reflectivity detection system (RDS) (**Figure 1B**), is a custom-made setup able to measure the reflectivity of growing photosynthetic cultures inside the ASC. It is composed of a removable custom-made baffling system hosting an optical fiber probe connected to a spectrometer (Flame, Ocean Optics). The baffling system has a circular hole of 30 mm through which part of the light coming from the light source can reach the growing culture. The design of the baffling system allows to leave only a little portion of the culture exposed to a collimated beam of light; to further improve the reduction of the stray light, the chamber is internally covered with a black coating for optical instruments (Spectral Black, Acktar) with a residual reflectivity in the visible spectral range of about ~ 2%. The setup is optimized for the reflectivity measurements by tuning the intensity of two LEDs before the collection of the data. Before the reflectivity measurement is acquired, light intensity of the 680 nm LED channel, in the VIS part of the spectra, and that of the 760 nm LED channel, in the NIR part of the spectra, are raised to improve signal to noise ratio and to match the spectrometer dynamic range with an optimal integration time. The light density reaching the culture during the reflectivity measurement is about 10 times lower than the growth light due to the baffling system, this is pivotal to avoid any damage to the growing cultures.

### Reflectivity and Normalized Difference Vegetation Indexes Measurements

The reflectivity of the cultures was assessed through the spectrometer using a custom-made script on MATLAB to collect and visualize data. For the reflectivity calculation, dark is subtracted and a white spectrum, with the same illumination and measurement integration time, is acquired before each experiment. Dark was taken at three different integration times (i.t.): 3, 4, and 20 s; the white was taken at an i.t. of 4 s for light intensities of 30 and 45 µmols of photons m^−2^s^−1^ and at an i.t. of 3 s for the light intensity of 95 µmols of photons m^−2^s^−1^, using support painted with a white optical coating placed on the bottom of the ASC. Cultures were always measured with an i.t. of 20 s, to allow an optimal signal to noise ratio for the following analyses. From the recorded reflectance spectra, the NDVI was calculated using a modified equation that considers narrow bands in the VIS and NIR instead of broad ones, as already explored by Gitelson and Merzlyak, 1994; Gamon and Surfus, 1999:

$$NDVI=\frac{\left(R_{745-755}-R_{675-685}\right)}{\left(R_{745-755}+R_{675-685}\right)}$$

R_745–755_: Reflectivity from 745 to 755 nm; R_675–685_: Reflectivity from 675 to 685 nm.

### Cultivation Conditions

*Synechocystis* sp. PCC6803 wt cells, from Pasteur Culture Collection (PCC, France), were grown in BG-11 medium (Rippka et al., 1979) and maintained in the exponential phase of growth by renewing the cultures with fresh medium each week. During growth, cells were exposed to atmospheric air and kept in a climatic chamber at 28–30°C under a continuous cool white fluorescent light of 30 µmols of photons m^−2^s^−1^ (L36W-840, OSRAM). For the experiments, cells in the exponential phase of growth were inoculated to an optical density of 0.2 at 750 nm in a final volume of 100 ml and grown as described before. On the day of the experiment, cells were renewed with fresh medium and brought to an optical density of 0.6 at 750 nm in a final volume of 100 ml. Forty microliters of culture were used for the validation experiments in the ASC, while the rest was used for biochemical analyses. Each growth experiment was carried on at least three biological replicates. In order to check the versatility of the RDS, the reflectivity spectra of several cultures of different cyanobacteria and microalgae species were collected. The cultures were comprised of the cyanobacteria *Synechocystis* sp. PCC6803, *Chlorogloeopsis fritschii* PCC6912, *Chroococcidiopsis thermalis* PCC7203, and *Arthrospira platensis* SAG85/79 grown in BG-11 medium and *Synechococcus* sp. PCC7335 grown in BG11+ASNIII (1:1 v/v) (Rippka et al., 1979). Microalgae cultures of *Chlorella vulgaris* CCAP221/11B, grown in BG11 medium and *Nannochloropsis gaditana* CCAP849/5 grown in f/2 medium were also tested (Guillard and Ryther, 1962). *A. platensis* was obtained from the Culture Collection of Algae at Göttingen University (SAG, Germany), while both microalgae strains were obtained from the Culture Collection of Algae and Protozoa (CCAP, United Kingdom).

### Dry Weight Determination

According to the measured optical density (OD), 10 or 15 ml of culture were diluted 1:3 with de-ionized water and filtered on 0.45 µm nitrocellulose filters (Sigma-Aldrich) using a vacuum flask. Filters were previously desiccated in a heater at 70°C for at least 3 h and weighted. Filters with cyanobacteria were put again in the heater at 70°C and left to desiccate; after at least 24 h they were weighted. Dry weight was measured as follows:

$$Dry\;Weight\;\left[\frac{g}{L}\right]=\frac{\left(Filter\;with\;cyanobacteria[g]-Empty\;filter[g]\right)}{Volume\;of\;culture\;filtered\;[mL]}\times 1000$$

### Chlorophyll a Quantification

For the extraction of Chlorophyll *a* from cell cultures, 2 ml of culture were centrifuged for 10’ at 10,000 x g. The supernatant was discarded and 1 ml of DMF (N,N′-dimethylformamide) was added to the pellet. Samples were kept at least for 24 h at 4°C in the dark, to allow the extraction of lipophilic pigments. Pigment concentration was assessed spectroscopically (Cary 300 UV-Vis, Agilent) using the Moran equation (Moran, 1982).

## Results

### Reliability of the Atmosphere Simulator Chamber and Alternative Configurations

The growth chamber (ASC, **Supplementary Figure 1**) was first verified to be able to maintain stable conditions over time monitoring delta pressure, temperature, CO_2_, and O_2_ (with maximum variations up to 1.8%). For the experiments with living organisms, the ASC was utilized in a configuration (**Figure 1A**) that allows the complete irradiation of the culture surface through the upper optical window, providing the energy supporting their growth. The periodic registration of the reflectivity spectra of the growing culture necessary for the determination of its NDVI was achieved by mounting on top of the upper window of the ASC the custom-made baffling system, embedding the optical fiber probe connected to the spectrometer (**Figure 1B**).

### Tracking by Remote Sensors Cyanobacteria Photosynthetic Gas Exchanges in Real-Time During Growth

**Figure 2:**
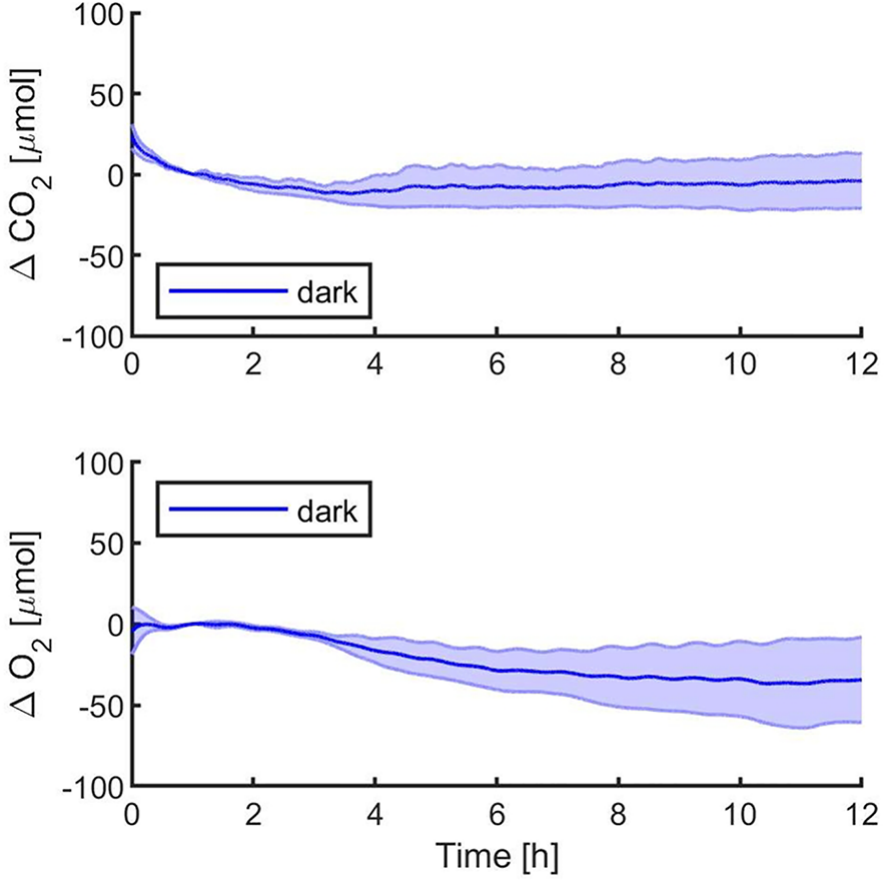
*Synechocystis* sp. PCC6803 carbon dioxide consumption and oxygen production over 12 h in the dark, with a starting atmospheric composition of air + 5%CO_2_. T_0_ was set at 3,600 s to exclude the initial gas equilibration period inside the chamber. Bold lines represent the average of three different biological replicates, with standard deviations reported as transparent areas.

**Figure 3:**
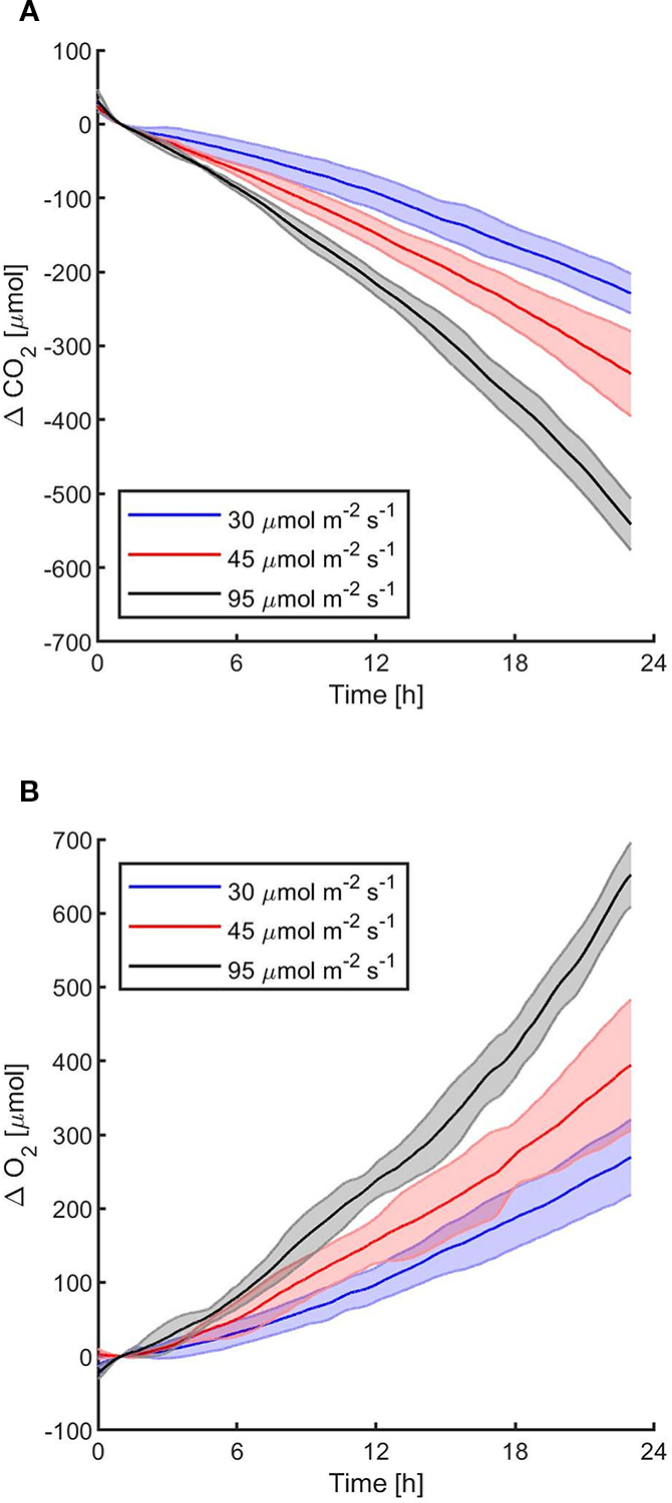
*Synechocystis* sp. PCC6803 carbon dioxide consumption **(A)** and oxygen production **(B)** over 24 h under three different light intensities, with a starting atmospheric composition of air + 5%CO_2_. T_0_ was set at 3,600 s to exclude the initial gas equilibration period inside the chamber. For each light intensity, the bold line represents the average of three different biological replicates, with standard deviations reported as transparent areas.

**Figure 4:**
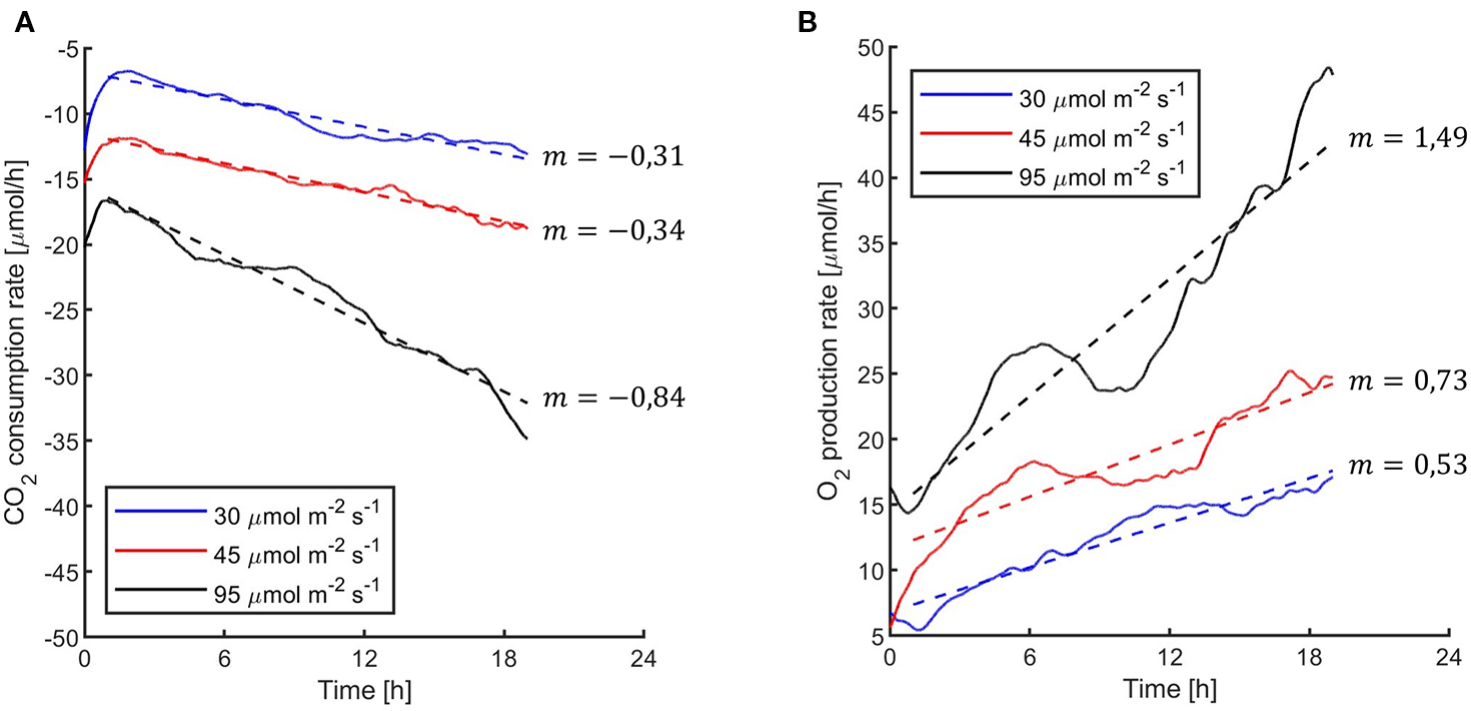
*Synechocystis* sp. PCC6803 carbon dioxide consumption rates per hour **(A)** and oxygen production rates per hour **(B)** under the three light intensities of the 24 h experiments reported in **Figure 3**. Data were obtained by calculating the second derivative of the curves in **Figure 3** over time. In the figure, it is reported the linear fitting for each light intensity.

**Figure 5:**
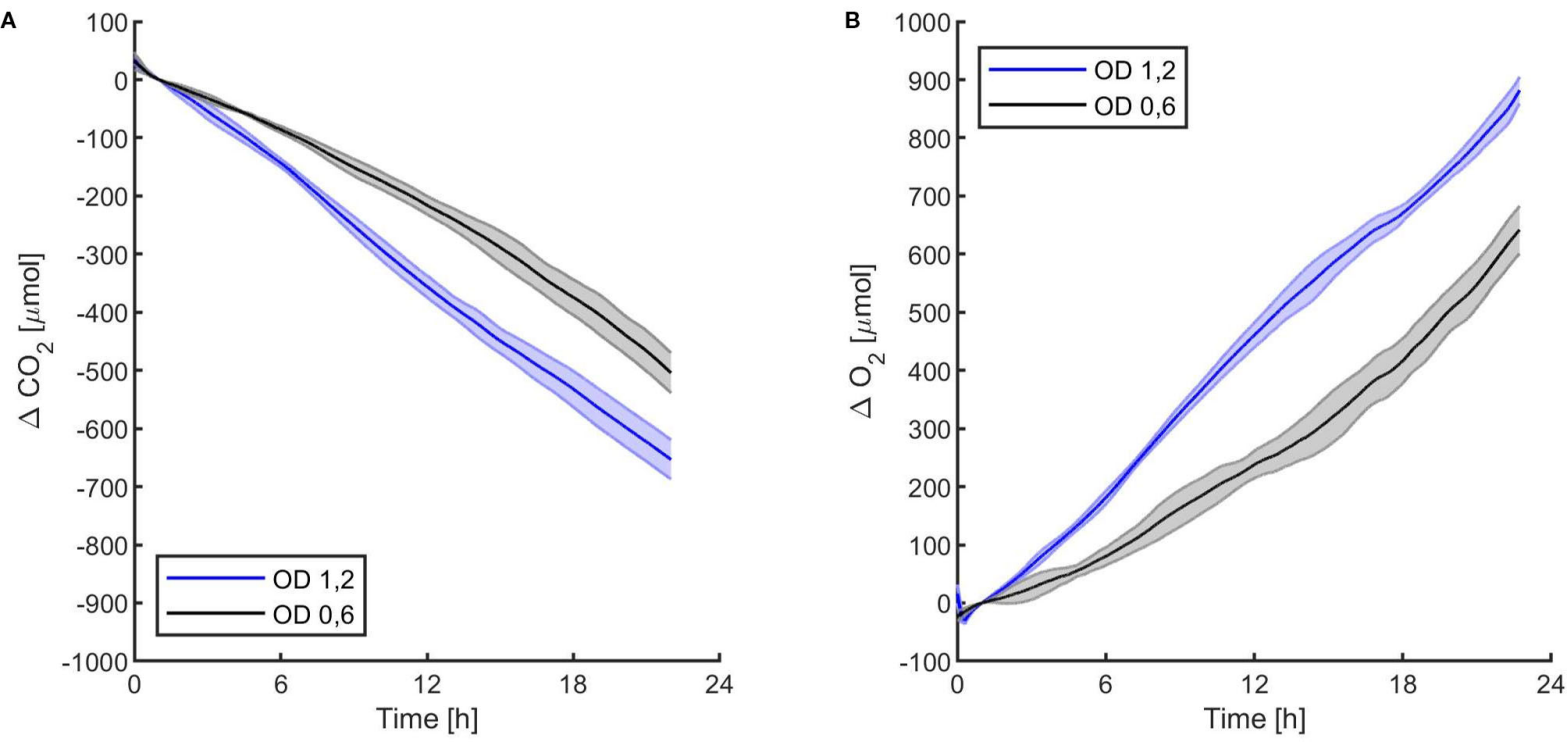
*Synechocystis* sp. PCC6803 carbon dioxide consumption **(A)** and oxygen production **(B)** over 24 h under 95 µmol of photons m^−2^s^−1^ of light intensity at two different optical densities, with a starting atmospheric composition of air + 5%CO_2_. T_0_ was set at 3,600 s to exclude the initial gas equilibration period inside the chamber. For each optical density, the bold line represents the average of three different replicates, with standard deviations reported as transparent areas.

The first experiments were focused on testing the setup potential for real-time and non-invasive detection of cyanobacteria photosynthetic responses to different light regimes. All experiments were carried out with *Synechocystis* sp. PCC6803 liquid cultures inside the ASC. At the beginning of each experiment, the ASC was filled with an atmosphere composed of 75%N_2_, 20%O_2_, 5%CO_2_ (called from now on “air + 5%CO_2_”). The temperature was set at 30 ± 0.5°C, 1 atm, and the culture was kept in the dark or illuminated with a solar light spectrum generated by the SLS (Salasnich et al., 2018) at three different light intensities of 30, 45, and 95 µmol of photons m^−2^ s^−1^. The changes in CO_2_ and O_2_ levels due to the photosynthetic activity of the cultures were recorded continuously during each experiment. Cyanobacteria cells kept in the dark show no photosynthetic activity, as expected, and a slight reduction in O_2_ and an increase of CO_2_, due to respiration (**Figure 2**). When cells are illuminated, instead, clear changes in O_2_ and CO_2_ concentration can be observed as a result of the photosynthetic activity, leading to O_2_ evolution and CO_2_ fixation into biomass. As seen in **Figure 3**, rates of O_2_ evolution/CO_2_ fixation depend on the light intensity, even between the two closest irradiances tested of 30 and 45 µmol of photons m^−2^ s^−1^, and this contributes to the different amounts of gasses exchanged. The graphs in **Figure 3** also highlighted that CO_2_ consumption and O_2_ evolution are not constant but follow a parabolic trend. This is likely because the photosynthetic activity increases with time due to cell duplication. The derivatives of gas exchange curves (**Figures 4A, B**) show that the growth trend depends on the light intensity and that cells exposed to the strongest light show the highest rate of increase, suggesting a faster growth rate. This indicates that continuous monitoring of CO_2_ consumption or O_2_ evolution can thus not only provide information on photosynthetic activities but also on culture growth. Data in **Figures 3** and **4** allow estimating the initial rates of CO_2_ consumption and O_2_ production per hour, as well as the total CO_2_ fixed into biomass and the total O_2_ released, as reported in **Table 1**. Information about growth rates, however, can be obtained only if the cultures do not absorb all available light. If cultures are more concentrated, in fact, cell pigments absorb all light energy available and thus cell duplication does not result in any increased activity since this is already maximal for those conditions. This is evident comparing the trends of CO_2_ and O_2_ concentration changes for culture at different OD exposed at the same light intensity (**Figures 5A, B**). At the higher OD, CO_2_ and O_2_ values follow a linear trend implying that the growth of the cells does not impact the photosynthetic rate. Despite this, it is still possible to have information on the initial photosynthetic activity by deriving the gas exchange curves.

**Table 1:** Initial rates of CO_2_ consumption, O_2_ production, and total µmoles of CO_2_ and O_2_ respectively fixed into biomass and released in the atmosphere in the three light intensities tested.

Light intensity (µmol m^−2^ s^−1^)	Initial rate of CO_2_ consumption (µmol h^−1^)	Initial rate of O_2_ production (µmol h^−1^)	µmoles of CO_2_ fixed in 24 h (µmol)	µmoles of O_2_ released in 24 h (µmol)
**30**	7.2	5.6	229.2 ± 27.0	269.5 ± 51.0
**45**	12.0	9.7	337.9 ± 57.5	394.4 ± 88.8
**95**	16.4	14.6	541.7 ± 34.7	652.2 ± 43.2

### Measurements of Cultures Reflectivity and Determination of Their Normalized Difference Vegetation Indexes as a Proxy of Growth

**Figure 6:**
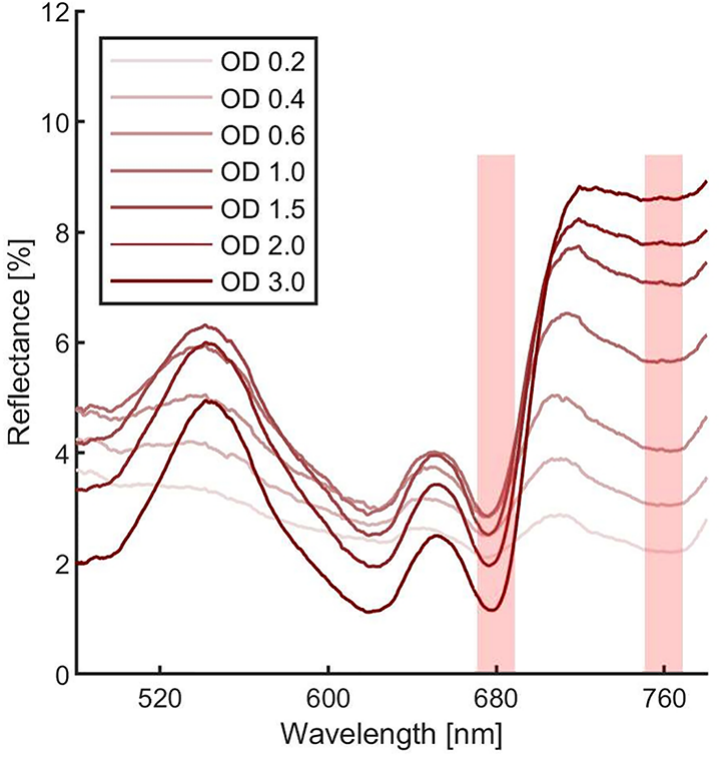
*Synechocystis* sp. PCC6803 reflectance spectra at different optical densities. Each measurement was averaged 10 times. Spectra were corrected for hot and dark pixels. Red shadowed rectangles represent were the Normalized Difference Vegetation Indexes (NDVI) can be calculated.

**Figure 7:**
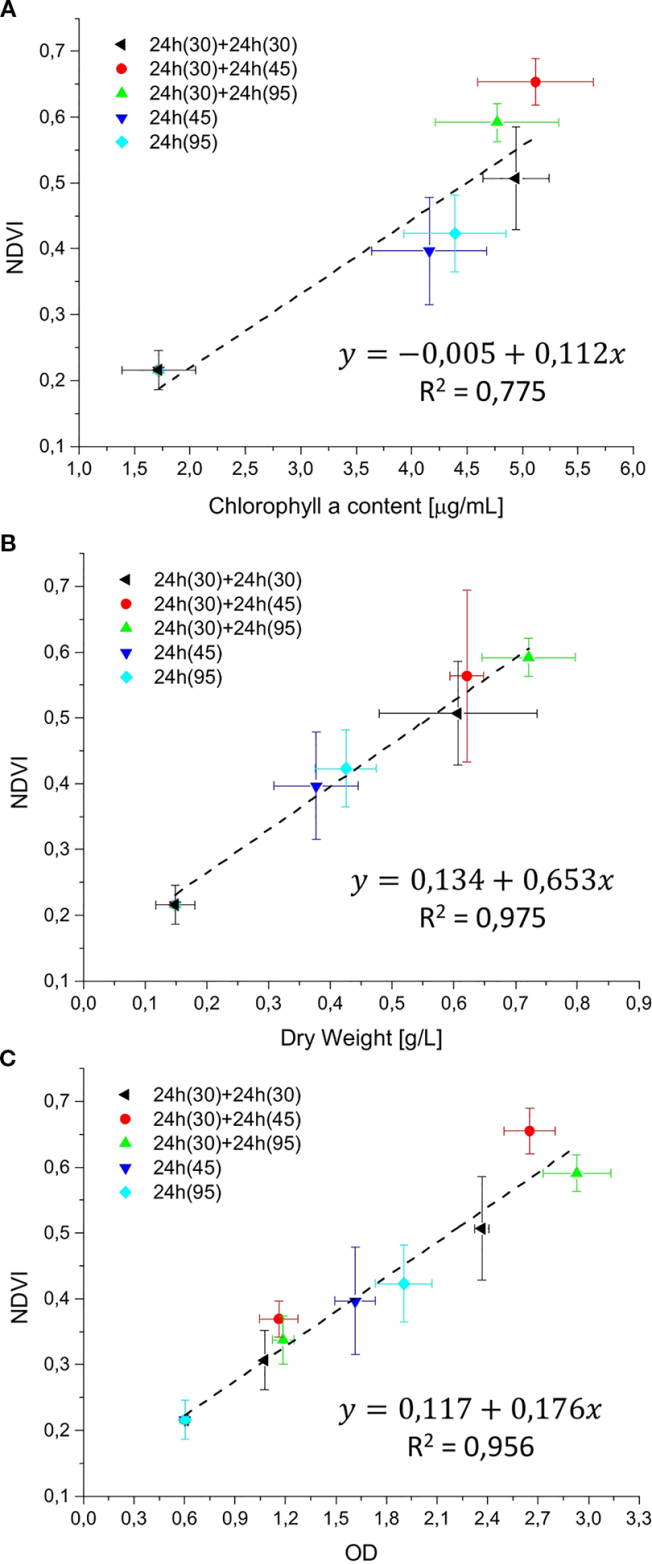
Reflectivity detection system (RDS) validation graphs. The NDVI was correlated with chlorophyll *a* content (µg/ml), dry weight (g/L), and optical density (OD), in **(A–C)**, respectively. Validation experiments were performed with *Synechocystis* sp. PCC6803 for 24 or 48 h, at light intensities of 30, 45, and 95 µmol of photons m^−2^s^−1^, with a starting atmospheric composition of air + 5%CO_2_. Black: 48 h at 30 µmol of photons m^−2^s^−1^; Red: 48 h experiment, combining 24 h at 30 µmol of photons m^−2^s^−1^ followed by 24 h at 45 µmol of photons m^−2^s^−1^; green: 48 h experiment, combining 24 h at 30 µmol of photons m^−2^s^−1^ followed by 95 µmol of photons m^−2^s^−1^; blue: 24 h experiment at 45 µmol of photons m^−2^s^−1^; cyan: 24 h experiment at 95 µmol of photons m^−2^s^−1^. For each type of experiment, three biological replicates were obtained. In experiments marked in black, red, and green, at 24 h the ASC was opened to collect a sample for the optical density (OD) measurement, then the atmosphere simulator chamber (ASC) was sealed again, and an atmosphere of air + 5%CO_2_ was re-filled inside the chamber.

**Figure 8:**
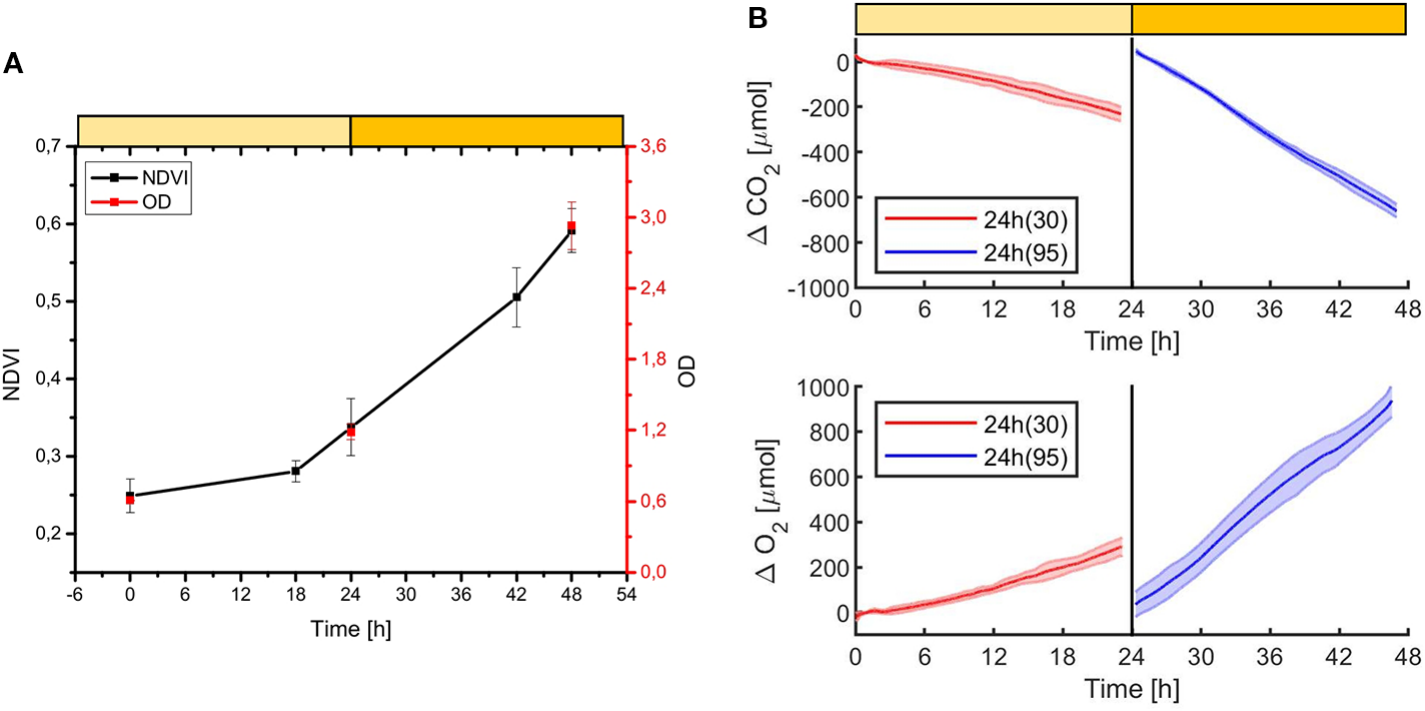
Example of *Synechocystis* sp. PCC6803 growth experiment monitored with the NDVI methodology **(A)**, and with the CO_2_ and O_2_ sensors **(B)**. The experiment was carried on for 48 h, the first 24 h at 30 µmol of photons m^−2^s^−1^, then at 95 µmol of photons m^−2^s^−1^ of light intensity, with a starting atmospheric composition of air + 5% CO_2_. At 24 h the atmosphere simulator chamber (ASC) was opened to collect a sample for the OD measurement, then the ASC was sealed again, and an atmosphere of air + 5%CO_2_ was re-filled inside the chamber. In **(A)** it is shown the growth over time of the OD (red dots) and the NDVI (black dots). In **(B)** it is shown the consumption of CO_2_ (upper box) and the production of O_2_ over time (lower box); in red are shown the first 24 h of the experiment at a light intensity of 30 µmol of photons m^−2^s^−1^, in blue the second 24 h, at 95 µmol of photons m^−2^s^−1^. T_0_ was set at 3,600 s to exclude the initial gas equilibration period inside the chamber. For each box, the bold lines represent the average of three different biological replicates, with standard deviations reported as transparent areas.

**Figure 9:**
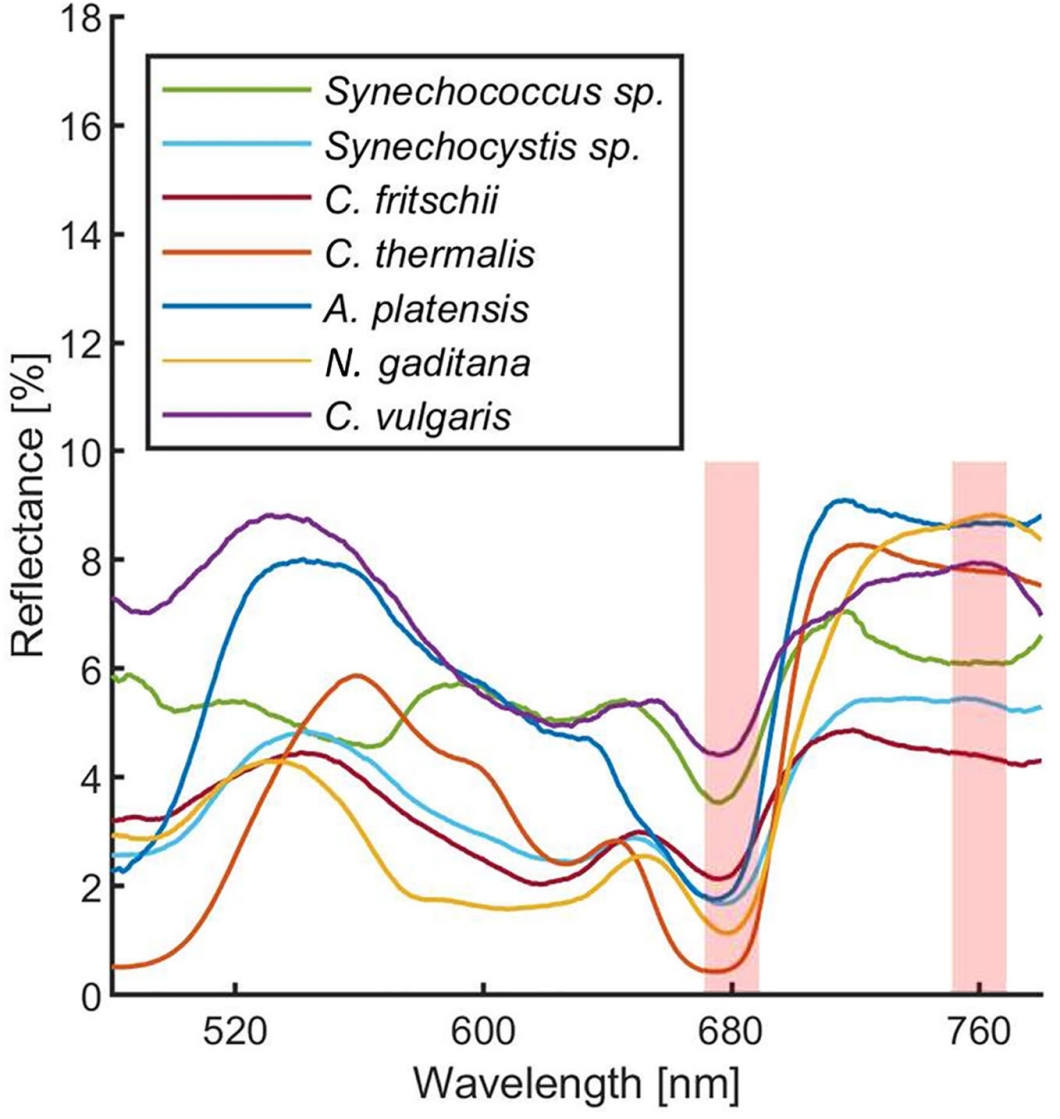
Reflectance spectra of different cyanobacteria and microalgae acquired with the RDS. Each measurement was averaged 10 times. Spectra were corrected for hot and dark pixels. Red shadowed rectangles represent were the Normalized Difference Vegetation Indexes (NDVI) are calculated. Green: *Synechococcus* sp. PCC7335; cyan: *Synechocystis* sp. PCC6803; Bordeaux: *Chlorogloeopsis fritschii* PCC6912; orange: *Chroococcidiopsis thermalis* PCC7203; blue: *Arthrospira platensis* SAG85/79; yellow: *Nannochloropsis gaditana* CCAP849/5; purple: *Chlorella vulgaris* CCAP221/11B.

In order to estimate the culture growth during long term experiments, when light or other factors become limiting, we applied a system based on remote reflectivity measurements, commonly employed by satellite or unmanned aerial vehicle remote sensors to evaluate the vegetation levels of our planet’s surface. To this aim, we designed and realized a custom-made setup (described in *Material and Methods*) for the detection of cultures reflectivity, to calculate their NDVI as a proxy of growth. Examples of reflectance spectra recorded by our setup from *Synechocystis* sp. PCC6803 liquid cultures at different OD are shown in **Figure 6**. To validate the reliability of the NDVI as a proxy of growth, we carried out a series of experiments growing the Cyanobacterium for 24–48 h in the ASC with a starting atmospheric composition of air + 5% CO_2_ and under different light regimes. The reflectance spectra and the relative NDVI were registered during each experiment while the OD, dry weight, and chlorophyll *a* concentration of the different cultures were determined at the beginning and at the end of the growth experiments. Correlations between the calculated NDVI and the measured growth parameters are plotted in **Figure 7**. Values of chlorophyll *a* content, dry weight, and optical density showed a linear relation with the NDVI values, demonstrating that this index is capable to assess the growth of the Cyanobacterium. This suggests the high potential of the RDS setup to evaluate the culture growth from remote. In **Figure 8A** an example of the NDVI validation experiments is presented. Here, cells were grown for 24 h at 30 µmol of photons m^−2^s^−1^ followed by another 24 h at 95 µmol of photons m^−2^s^−1^. The NDVI was calculated multiple times during the experiment, while the OD was measured at t_0_ (0 h) and at t_f_ (48 h), as well as after 24 h, by opening the ASC. The NDVI and the optical density showed the same trend over time, also highlighting a difference in the growth rate according to the increase of light intensity. This well correlated with the changes in CO_2_ and O_2_ levels upon the change of light where it was observed a 3-fold increase in the amount of CO_2_ fixed and O_2_ released at the end of the experiment respect to the first 24 h (**Figure 8B**). Finally, with the RDS, we registered the reflectance spectra of different cyanobacteria and microalgae species (**Figure 9**). We assessed three cyanobacteria species (*C. fritschii* PCC6912, *C. thermalis* PCC7203, *Synechococcus* sp. PCC7335), and the already utilized Cyanobacterium for BLSS (*A. platensis* SAG85/79) as well as the microalgae *Chlorella vulgaris* CCAP221/11B and *Nannochloropsis gaditana* CCAP849/5, considered promising candidates for BLSS and high-value lipid production respectively. The comparison of the different reflectance spectra with that of *Synechocystis* sp. PCC6803 indicates that for each organism, specific correlation experiments are needed in order to utilize the NDVI as a precise method for their growth quantification. Even in the absence of such preliminary information, however, NDVI measurements still can provide valuable semiquantitative information since its increase would anyhow evidence growth even if not precisely quantified.

## Discussion

The future of space and planetary missions requires innovative technologies that must be developed on the basis of preliminary experiments performed in laboratory facilities simulating specific environmental conditions. Oxygenic photosynthetic microorganisms have a key relevance in both bioregenerative life-support systems but also in evaluating the habitability of planetary bodies and thus there is in the field the strong necessity of investigating their physiological responses to beyond-Earth physico-chemical parameters. Small scale simulation chambers capable of generating different light regimes, atmospheric compositions, temperatures, and pressures, to simulate different environmental conditions are essential tools to achieve this goal. Such chambers should also allow easy monitoring of microorganisms’ survival, growth, and gas exchange responses. Several simulator chambers have been realized and utilized so far for the simulation of Mars and other beyond-Earth environments (Tarasashvili et al., 2013; Mateo-Marti, 2014), with few of them also able to test aqueous cultures with metabolically active organisms (Martin and Cockell, 2015). Most of these simulator chambers, however, are complex facilities that do not allowing organism’s growth but only to test their survival under non-terrestrial conditions, with the possibility of evaluating physiological responses only after the experiments. The system presented here (**Supplementary Figure 1**) permits control of pressure, temperature, and atmospheric gas composition (at least with N_2_, CO_2_, and O_2_) while allowing the growth of photosynthetic microorganisms under the selected conditions. The geometry of the ASC is a further added value since it allows homogeneous irradiation of the samples lodged in the petri dish containing the organisms on liquid or solid media, minimizing cells self-shading. Another value is the culture volume that, despite being lower than that of some other available chambers (Martin and Cockell, 2015), still allows us to obtain a sufficient amount of biomass to perform biochemical, physiological, and omics analyses on acclimated cells at the end of the experiments. Finally, the setup allows monitoring the microorganism’s physiological responses in a non-invasive way. Photosynthetic gas exchanges are measured in continuous by remote sensors without the need for sampling the cultures. This avoids introducing any alteration of the environmental parameters set and analyzing microorganism’s photosynthetic performances under conditions different from the desired experimental ones. The continuous monitoring of gas levels increases the reliability of the setup evidencing variations due to slight changes in light intensity (**Figure 3**). The gas level curves, furthermore, can be highly informative, providing information on the photosynthetic capacity of the microorganisms but also on the growth rate (**Figure 4**), if the cell concentration is low enough (**Figure 5**). This further improves our ability to collect information on the behavior of the photosynthetic organisms when directly exposed to the experimental conditions. Additionally, we have also been able to remotely monitor the reflectance spectra of microorganism’s liquid cultures maintained in a sealed environment and to determine their NDVI to follow their growth (**Figures 6** and **7**). This was achieved with the RDS, by adapting at lab scale the method so far used to detect photosynthetic microorganism blooms in natural environments (Oyama et al., 2015; Teta et al., 2017). Several portable NDVI detection devices are present in the market but are designed for plant leaves (Yu et al., 2016), thus they were unsuitable for our purposes. Other equipment utilizes specific cameras to obtain VIS/NIR images or to monitor the reflectance spectra of photosynthetic organisms present in the environment (Yu et al., 2016). However, these systems are inapplicable for lab-scale microorganism’s cultures as they need to be positioned at a certain distance and with a determined angle from the samples and are strongly influenced by the presence of reflective surfaces such as metal or glass components of the growing chambers. The custom-made setup that we developed overcomes all these issues allowing reliable detection of culture reflectivity (**Figure 1**). The optical fiber allows to reduce significantly the size of the device and the designed baffling system (where the optical fiber is lodged) together with the internal black coating of the ASC allow precise measurement of the reflectivity of the culture, free from any stray light. The measurement of the NDVI parameter results of high importance for long term growth experiments, when operational parameters such as light or nutrients become limiting for the growth and gas level changes can’t be informative anymore. Different cyanobacterial and microalgal strains show different reflectance spectra when measured with the RDS (**Figure 9**), indicating that is mandatory to perform a calibration for each species of interest. The differences in the spectra indeed depend upon the pigment composition of each strain, the geometry and dimensions of the cells as well as their responses to environmental conditions (Kiang et al., 2007). Even if a direct quantification of the growth is possible only performing for each microorganism a calibration that correlates the NDVI changes with biomass concentration, the increase of NDVI value over time still provides a reliable indication of cells growth. Combining the SLS capacity to simulate different emission spectra of stars to the RDS it will be possible to detect in the tested microorganisms the so-called “red edge”. This is a spectral feature that appears as a bump in the reflectivity beyond 700 nm, typical of all photosynthetic oxygenic organisms (Kiang et al., 2007), that has become the most promising surface biosignature to search for oxygenic life on remote exoplanets. Recently, many Earth-like exoplanets have been found orbiting M-dwarf stars, with notable examples being the TRAPPIST-1 planet system and Proxima Centauri b (Anglada-Escudé et al., 2016; Gillon et al., 2017). As these stars are characterized by a low emission in the visible and larger contributions in the far red and infrared, compared to the Sun, it is still under debate if the adaptation to such a light spectrum could trigger a shift in the red edge (Takizawa et al., 2017) generated by photosynthetic organisms. With our setup, it will be possible to assess the response of various terrestrial organisms to this simulated illumination. During these experiments, combining the SLS with the ASC, it will be also possible to test the oxygenic photosynthetic organisms’ performances and to analyze their impact on different atmospheric compositions, such as primeval ones, lacking oxygen. This facility represents a new frontier in simulation and remote sensing detection, and it could contribute to define the physico-chemical limits of oxygenic photosynthesis of terrestrial organisms and their physiological responses to beyond-earth conditions. Data on growth and gas exchange rates, biomass production, will be seminal for the development of bioregenerative life support systems. Final aims in the field of space exploration will be those of producing surface and atmospheric biosignature databases, that will be an important reference when future space missions will begin to deliver atmospheric composition and surface reflectance data about extrasolar planets.

## Data Availability Statement

The datasets generated for this study are available on request to the corresponding author.

## Author Contributions

RC, LP, LC, and ME conceived and realized the Star Light Simulator (SLS) and the Atmosphere Simulator Chamber (ASC). BS designed the SLS control software. BS and EA calibrated the SLS. NR, LC, and MB conceived the Reflectivity Detection System (RDS). LC realized it. MB, NR, and TM conceived the biological experiments. MB performed them and analyzed the data. LC, TM and NR contributed to the analyses. NR wrote the manuscript. MB, LC, and TM edited it while LP and CR critically read it.

## Funding

The research was co-funded by the Italian Space Agency through the “Life in Space” project (ASI N. 2019-3-U.0), and by the Department of Biology of University of Padova and the Institute for Photonics and Nanotechnologies of CNR through intramural grants.

## Conflict of Interest

The authors declare that the research was conducted in the absence of any commercial or financial relationships that could be construed as a potential conflict of interest.
