# Supplementary material for: A New Remote Sensing-Based System for the Monitoring and Analysis of Growth and Gas Exchange Rates of Photosynthetic Microorganisms Under Simulated Non-Terrestrial Conditions
Source: Front Plant Sci. 2020 Mar 4;11:182. doi: 10.3389/fpls.2020.00182 (PMC7066451; doi:10.3389/fpls.2020.00182)
Supplement: Supplementary file 1 [file Table_1.docx]

Supplementary Material


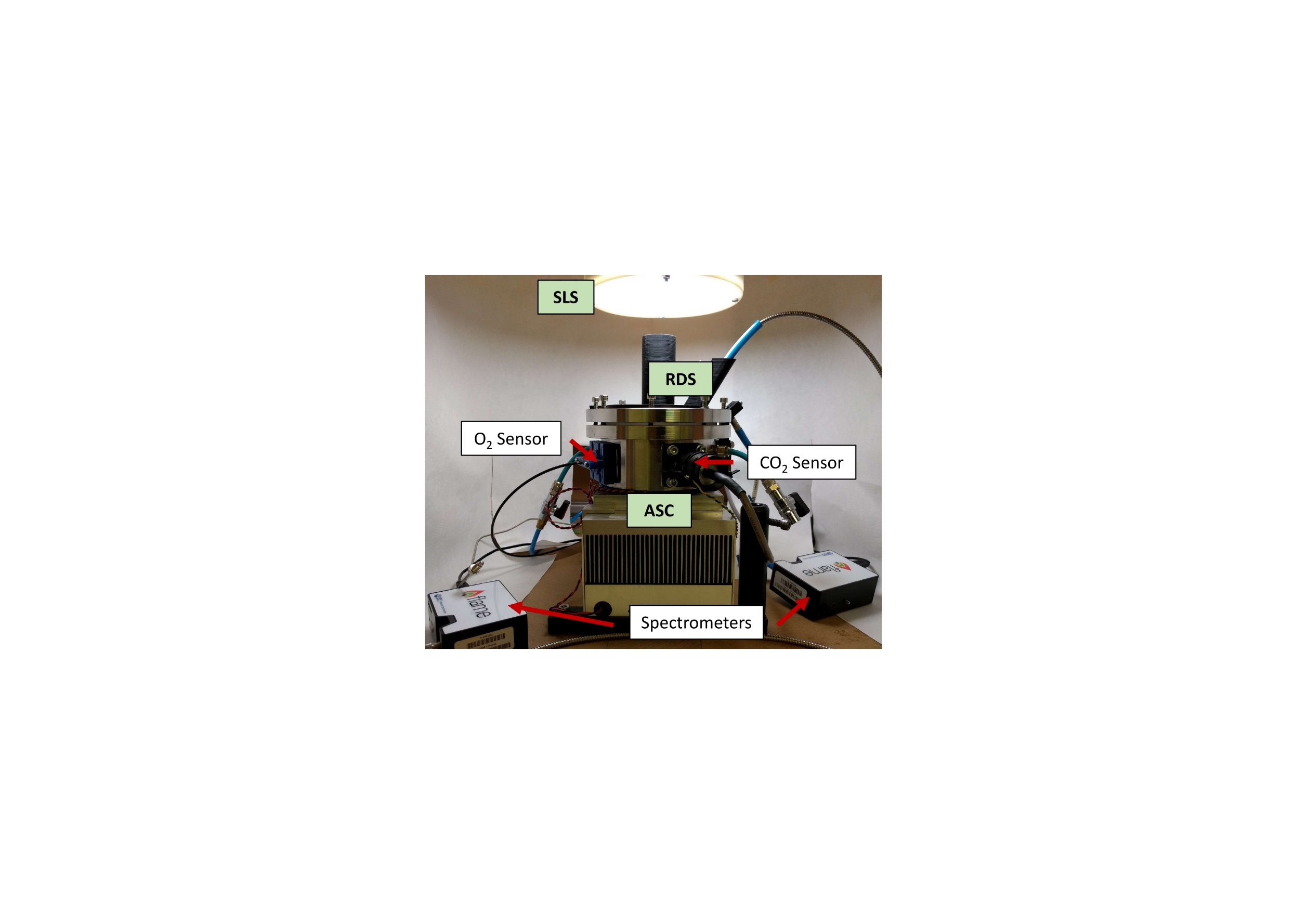


**Supplementary Figure 1.** Picture of the ASC-SLS-RDS setup. CO_2_, O_2_ sensors and the two spectrometers (one for the SLS, the other for the RDS) are highlighted
